# Designing Toxicogenomics Studies that use DNA Array Technology

**Published:** 2008-08-14

**Authors:** Robert R. Delongchamp, Cruz Velasco, Varsha G. Desai, Taewon Lee, James C. Fuscoe

**Affiliations:** 1 Biometry Branch, Division of Personalized Nutrition and Medicine, National Center for Toxicological Research, 3900 NCTR Road, Jefferson, AR 72079; 2 Department of Epidemiology, Fay W. Boozman College of Public Health, University of Arkansas for Medical Sciences, Little Rock, AR 72205; 3 Louisiana State University Health Sciences Center, New Orleans, LA 70112; 4 Center for Functional Genomics, Division of Systems Toxicology, National Center for Toxicological Research, 3900 NCTR Road, Jefferson, AR 72079

## Abstract

**Background:**

Bioassays are routinely used to evaluate the toxicity of test agents. Experimental designs for bioassays are largely encompassed by fixed effects linear models. In toxicogenomics studies where DNA arrays measure mRNA levels, the tissue samples are typically generated in a bioassay. These measurements introduce additional sources of variation, which must be properly managed to obtain valid tests of treatment effects.

**Results:**

An analysis of covariance model is developed which combines a fixed-effects linear model for the bioassay with important variance components associated with DNA array measurements. These models can accommodate the dominant characteristics of measurements from DNA arrays, and they account for technical variation associated with normalization, spots, dyes, and batches as well as the biological variation associated with the bioassay. An example illustrates how the model is used to identify valid designs and to compare competing designs.

**Conclusions:**

Many toxicogenomics studies are bioassays which measure gene expression using DNA arrays. These studies can be designed and analyzed using standard methods with a few modifications to account for characteristics of array measurements, such as multiple endpoints and normalization. As much as possible, technical variation associated with probes, dyes, and batches are managed by blocking treatments within these sources of variation. An example shows how some practical constraints can be accommodated by this modelling and how it allows one to objectively compare competing designs.

## Background

Many toxicogenomics studies are intended to evaluate the response of the transcriptome to test agents. Observed changes in mRNA concentrations in tissues can indicate the mechanism of toxicity, at least in concept (Chin and Kong, 2002; Guerreiro et al. 2003; Hamadeh et al. 2002; Irwin et al. 2004; Lord et al. 2006; Luhe et al. 2005; Nuwaysir et al. 1999; Waring and Halbert, 2002; Waters et al. 2003). However, the analysis and interpretation of these studies confront many problems that are often compounded by poor designs (Lay et al. 2006).

The response of an organism to applied treatments or selected characteristics is evaluated in a bioassay. The bioassay, that underlies most toxicogenomics studies conducted at the National Center for Toxicological Research, is to treat animals of specified phenotypes (e.g. species, sex, strain) or conditions (e.g. ages, calorie restricted) with various levels of test agents (e.g. doses, exposure regimes). The study designs are typical of bioassays routinely used in toxicology. In fact, tissues are often collected from bioassays primarily designed for more traditional purposes. Many design issues, such as sample size or power, can largely be addressed through standard procedures for multi-factor designs. The goal in this paper is to describe a heuristic model for the statistical analysis that integrates the usual approach to designing bioassays with the added complications associated with measuring expression with DNA arrays.

DNA array technologies simultaneously measure transcript levels from thousands of genes per sample, and this ability to interrogate transcript levels for an entire genome motivates the interest in toxicogenomics studies. However, these measurements are generated through chemical assays comprised of several steps, which are prone to technical variation (Yang and Speed, 2002). The study design must incorporate these technical sources of variation with the biological variation in the bioassay in a way that does not compromise treatment comparisons.

The data generated in these studies are analyzed ‘by gene’, and the interpretation of the significance of p-values associated with tests for treatment effects must cope with misclassification rates expected with such a large number of tests. In this setting, it is desirable to declare significance based on false discovery rates (Allison et al. 2006; Benjamini and Hochberg, 1995; Delongchamp et al. 2004). The traditional power/sample size calculation for a single test remains useful to evaluate the relative merits of competing designs, but benefits from modifications, which arise by consideration of multiple testing.

It is common practice to transform, normalize and background correct the intensities from DNA arrays although there is no widespread agreement on the best procedures (Allison et al. 2006). Each procedure impacts the analysis, and this should be addressed in the design. While we have no special insight as to the best procedures, the specific procedures, which we use, are justified in the context of an overall analysis strategy. For actual examples of analyses, see (Delongchamp et al. 2003; Delongchamp et al. 2005; Desai et al. 2007; Desai et al. 2004).

Hypotheses are tested using an analysis of covariance model to account for the variance removed by normalizing the measurements and to adjust for specified sources of variation (Parrish and Delongchamp, 2006). These designs often use a standard reference sample (Desai et al. 2007; Desai et al. 2004). While a ‘reference’ design can be less efficient than balanced incomplete block designs (Kerr and Churchill, 2001; Vinciotti et al. 2005), they offer versatility with respect to treatment layouts and sample sizes. A recent set of papers discusses some of these issues (Dobbin et al. 2003b; Dobbin et al, 2005; Martin-Magniette et al. 2005a; Martin-Magniette et al. 2005b). From our perspective, the goal is to design studies which reliably test treatment effects with adequate power, and the designs we use meet this goal. Designs, which estimate expression changes relative to a standard, facilitate across-study comparisons. Since functional genomic studies tend to be exploratory, follow-up studies should be anticipated and estimating effects relative to a standard has merit in this context.

## Methods

At the design stage, a statistical model is a framework through which experimental design questions can be addressed. Our goal is to design a toxicogenomics study that allows valid treatment comparisons when confronted by several sources of variation. Given a design, the model allows one to incorporate the recognized sources of variation into expected mean squared errors for a statistical analysis. Before a design is implemented, its expected mean squares should demonstrate that an analysis with valid tests is possible. Usually several designs can give valid tests, and the model helps to choose among them. An example illustrates the design process.

### Model for bioassay

It will be taken for granted that tissue samples and treatments have been suitably selected to address hypotheses of interest. For didactic purposes, we presume that the treatment layout of the bioassay is a fixed effects linear model as this level of generalization accommodates the majority of toxicogenomics studies conducted at NCTR. Under a fixed effects linear model, the ‘expression’ data for an arbitrary gene has an expectation and variance that can be written as

(1.1) $$\begin{array}{l} E(y_{i})=(X_{i1}\ldots X_{ip})\beta =X_{i}\beta \\ \text{var}(y_{i})=\sigma^{2} \end{array}$$

At this juncture, *y_i_* denotes an observed measurement of expression, suitably transformed, for experimental unit, *i*, obtained at treatment levels specified through a design matrix, **X***_i_*, and *σ*^2^ denotes the within-treatment variance among experimental units.

Equation (1.1) models an arbitrary gene. To “scale up” to the thousands of genes which are measured per experimental unit, the parameters, **β** and σ^2^, are assumed to be gene specific. The multivariate structure is ignored in practice, and statistical analyses are applied one gene at a time (Delongchamp et al. 2004; Wolfinger et al. 2001). Consequently, Equation (1.1) is adequate for this discussion. From the computation perspective, fixed effects linear models are widely implemented in well documented statistical packages. Our experience is with SAS, and these models are easily implemented using PROC GLM (SAS, 1999). Further, this basic design structure can accommodate more sophisticated analyses, which incorporate current trends in statistical methods for DNA array data (Allison et al. 2006). It is not too difficult to implement shrinkage/empirical Bayes adjustments to the variance (Smyth, 2004; Wright and Simon, 2003), which will increase statistical power when sample sizes are small. We have also adapted these analyses to correlated groups of genes (e.g. gene ontology groupings) (Delongchamp et al. 2006).

Without further elaboration, we presume that the set of biological samples, {**X***_i_*: *i =* 1,…,*n*}, defines a reasonably efficient design with respect to the hypotheses of interest, and note that the properties of experimental designs have been well studied in this setting. In our work, ‘*n*’ is arrived at through a conventional sample size/power assessment, which addresses the issue of multiple testing by reducing the significance level, e.g. power is computed at significance level of 0.001 instead of 0.05 for a representative value of *σ*^2^. This is elaborated through the example in Section 5.

### Model for DNA arrays

For our purposes, a DNA array is a surface that is spotted with DNA. The spots are arranged in a grid at a regular spacing, the array. Each spot is DNA with a known nucleotide sequence that corresponds to part of a gene’s DNA sequence. Hereafter, the spotted sequence will be referred to as a probe. When a mixture of DNA species is hybridized to the arrayed probes, complementary sequences bind preferentially thereby sorting the mixture into its constituents. To measure a sample, which is a mixture of mRNA species, an aliquot of the total mRNA is transcribed into complementary DNA. This cDNA is labelled with a florescent dye or a radioactive isotope. The labelled-cDNA is hybridized to the probes, and the intensity of label bound to each probe is measured. More than one sample can be hybridized to an array by using different labels.

There are several commercially available platforms that measure gene expression by hybridizing a labelled sample to a set of DNA probes, and their details can differ from the preceding description. Our experience has been with spotted arrays, both ‘macro’ arrays, e.g. www.clontech.com (Bowyer et al. 2007; Delongchamp et al. 2003; Delongchamp et al. 2005) and oligonucleotides spotted on glass-slide microarrays, such as ‘Mitochip’ (Desai et al. 2007). Because of our experience, we describe the statistical model in the context of a spotted oligonucleotide technology, specifically ‘Mitochip’. In large part, the statistical issues do not depend on how a DNA probe is created. That is, *in situ* synthesized microarrays, such as arrays manufactured by Affymetrics: www.affymetrix.com, record a label-intensity at each ‘feature’, which has essentially the same statistical properties as the spotted technologies. Affymetrics arrays measure multiple probes for each gene, both sequence matches and mismatches. To obtain a single expression measurement, it is necessary to collate intensities from many probes. Likewise, it is not necessary that the DNA probes are physically bound to a surface. For example, the Illumina bead platform, www.illumina.com, still obtains label-intensities associated with a set of DNA probes even though the beads are not arrayed. While PCR methods are technologically distinct, the modeling employed here extends quite easily to that technology (Desai et al. 2007).

Let 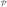 denote the set of probes on an array. Label intensities are observed at the location of each probe. These intensities need to be related to the mRNA concentrations in the original tissue. In general, a tissue sample can be assayed more than once. To accommodate this, we add a subscript to designate the aliquot measured on an array. The intensity observed at a probe (*g*) for an aliquot/array (*a*) from experimental unit (*i*) will be designated *I_ga_*_(_*_i_*_)_.

In evaluating a gene’s function, we attempt to observe how its mRNA concentration responds to manipulated conditions. The concentration of mRNA for gene (*g*) in a sample of tissue (*i*) will be denoted as *C_gi_*. It is usually assumed that the intensity is proportional to the mRNA concentration in the sample, *I_ga_*_(_*_i_*_)_ ∝ *C_gi_*. In general, this assumption is not correct unless background contributions to the intensity can be ignored and probe amounts are in excess of amounts in the aliquot hybridized to the array (Held et al. 2003; Held et al. 2006; Zhang et al. 2007). These issues are limitations of the technology that are not directly addressed by the design considerations discussed here.

For two samples, no difference in the expression of a gene is taken to mean that the mRNA concentrations are equal, that is *C_gi_* = *C_g j_*. The total concentration of mRNA in a sample is the sum of concentrations from the constituent mRNAs (genes). Since *C_gi_* = 0 when a mRNA species is absent, the total concentration can be written as the sum over genes that comprise the genome. Let 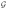 denote this set, and write this sum as

(1.2) $$C_{•i}=\sum_{g\in G}C_{gi}.$$

Then the proportion of the total mRNA that arises from gene (*g*) is

(1.3) $$\pi_{gi}=C_{gi}/C_{•i}.$$

An assay of gene expression begins with a fixed amount (*w* μg) of the total mRNA, which was recovered from a tissue sample, so that *π_gi_* *w* μg of mRNA from gene ‘*g*’ is in the starting aliquot. Note that equal amounts of a specific mRNA in two starting aliquots do not imply that the initial samples have equal expression since *π_gi_* *w* = *π_gj_* *w* does not imply that *C_gi_* = *C_gj_*. For equal amounts in aliquots to imply equal concentrations in samples, the total mRNA must be the same for both samples, that is *C*_•_*_i_* = *C*_•_*_j_*. This usually would not be the case if some genes in these samples have differential expression. In order to ‘normalize’ the amounts in aliquots so that they reflect the concentrations in tissues, one needs to know or estimate the ratio, *C*_•_*_i_*/*C*_•_*_j_*.

The essential chemistry is the specificity of DNA hybridizations. For every probe on the array, *g* ∈ 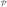, and gene in the genome, *h* ∈ 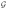, there is a binding affinity, κ*_gh_*. If the gene-specific amounts of labelled cDNA are proportional to the corresponding mRNA in the aliquot, the intensity will be proportional to the relative concentration of gene ‘*g*’, κ*_gg_* *π_gi_*, plus a background, ∑*_h_*_∈ 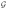
_*_, h_*_≠_*_g_* *κ_gh_π_hi_*, which arises from binding with DNA from other genes in the sample. That is,

(1.4) $$I_{ga(i)}\propto w\sum_{h\in G}\kappa_{gh}\pi_{hi}=w\left(\kappa_{gg}\pi_{gi}+\sum_{h\in G,h\neq g}\kappa_{gh}\pi_{hi}\right).$$

Successful sorting implies that the binding to probe ‘*g*’ is much larger for labelled cDNA from gene ‘*g*’ than for labelled cDNA from any other gene, i.e. *κ*_gg_ ≫ *κ_gh_* when *h* ≠ *g*. Since the background should be small, it is of little consequence when the interrogated gene is highly expressed, in which case: *I_ga(i)_* ∝ *π_gi_*. Background is problematic when *π_gi_* is also small. At least for this discussion, this problem with background is viewed as a limitation of the technology that biases estimated effects.

As an operational model, we ignore background and assume that the observed intensity is reasonably approximated as

(1.5) $${\text{log}}_{2}I_{ga(i)}\approx K_{ga}+{\text{log}}_{2}C_{gi}-{\text{log}}_{2}C_{•i}.$$

The observed log-intensity depends upon mRNA concentration in the sample, log_2_ *C_gi_*, as well as a proportionality term associated with processing the aliquot, *K_ga_*, and a ‘normalizing’ term, log_2_ *C*_•_*_i_*.

### Technical variation: spot, dye and batch effects

Whenever two samples are hybridized to a single array, they share some sources of variation. In particular, hybridization conditions, DNA binding sites, and spot alignments will be identical. Common conditions make intensities for these samples more alike than those from samples that have been measured on other arrays. That is, samples measured on different arrays exhibit more technical variation, which we refer to as a spot effect. In order to hybridize two samples on an array, they must be labelled with different dyes. There also is a dye effect. Spot and dye effects can be directly estimated by a dye-flip between two samples (Chen et al. 2004).

Consider two samples, *i* and *j*, which are measured with a dye flip on two arrays. Table 1 illustrates the layout, where we let *y* = log_2_ *I* to streamline the notation. By measuring each sample twice, the array/spot effect and the dye effect are estimable and they can be eliminated from the estimate of the treatment effect. In this context, the dye effect is defined to be the difference between the average of intensities for the respective dyes, i.e.

$$2d=y_{11}+y_{12}-y_{21}-y_{22}.$$

Similarly, the array effect, treatment effect, and overall mean are defined as

$$\begin{array}{l} 2a=y_{11}-y_{12}+y_{21}-y_{22}\\ 2t=y_{11}-{\text{y}}_{12}-y_{21}+y_{22}\\ 4m=y_{11}+y_{12}+y_{21}+y_{22}. \end{array}$$

At least heuristically these equations can be related to Equation 1.5 as follows:

$$\begin{array}{l} y_{11}=(m+t/2)+(a/2+d/2)\approx {\text{log}}_{2}\pi_{i}+K_{11}\\ y_{12}=(m-t/2)+(-a/2+d/2)\approx {\text{log}}_{2}\pi_{j}+K_{12}\\ y_{21}=(m-t/2)+(a/2-d/2)\approx {\text{log}}_{2}\pi_{j}+K_{21}\\ y_{22}=(m+t/2)+(-a/2-d/2)\approx {\text{log}}_{2}\pi_{i}+K_{22} \end{array}$$

That is, the proportionality constant in Equation 1.5 depends on spot and dye effects which can be estimated and eliminated via this dye flip. In particular,

$$t={\text{log}}_{2}\pi_{i}-{\text{log}}_{2}\pi_{j}=(y_{11}+y_{22})/2-(y_{12}+y_{21})/2.$$

A dye-flip works well when there are two treatments since the treatments can be blocked within spots and dyes (Delongchamp et al. 2005). However, it becomes increasingly difficult to manage technical sources as the number of treatments is increased. A straightforward solution is to do the dye flip with a standard sample, e.g. (Desai et al. 2004), where the standard is characterized as a sample which has a constant expression level throughout the study.

A dye-flip with a standard uses two arrays per sample and doubles the cost and work of assaying each sample. However, each sample is measured twice, thereby reducing the technical error. This can be a reasonable way to increase the precision of estimated treatment effects whenever tissue samples are at a premium. At least conceptually, this type of dye flip eliminates spot effects and dye effects yielding adjusted estimates of expression in the samples relative to expression in the standard. In earlier work, there was an interest in mining databases created from several studies, and estimates of expression relative to a common standard seemed desirable. In practice, the sequences used for probes, assay conditions, etc., change from study to study and these changes undermine the utility of measuring expression relative to a standard.

In current work, we usually label the sample with CY5 and a universal reference with CY3. The difference between the log-intensities of the sample and reference eliminates the spot effect but not the dye effect, *y*_11_ − *y*_21_ = *t* + *d*. The dye effect usually differs for each probe. Within a probe, this effect appears to be reasonably stable across samples, and in recent studies we presume it is a constant bias in the difference between the sample and the standard. Because we block on batches, a dye ‘bias’ only needs to be constant within batches to manage this effect.

In addition to variation associated with spots and dyes, we explicitly design studies to manage technical sources of variation that can be associated with concurrent processing conditions (batches). Samples assayed concurrently share processing conditions making their measurements more homogeneous than measurements made at other times. Some of these sources of variation may not be trivial resulting in a ‘batch’ effect. In general, all of the samples in a study cannot be processed concurrently. So, the batch effect should be incorporated into the experimental design.

### Combined model

Let *y_gi_* be a difference between a sample and the standard,

$$y_{gi}={\text{log}}_{2}I_{ga(i)}-{\text{log}}_{2}I_{ga(s)}.$$

This gives

(1.6) $$\begin{array}{l} \text{E}[y_{gi}]\approx \text{E}[d]+{\text{log}}_{2}(C_{gi}/C_{gs})-{\text{log}}_{2}(C_{•i}/C_{•s})\\ =\text{E}[d]+X_{i}\beta_{g}-{\text{log}}_{2}(C_{•i}/C_{•s}) \end{array}$$

To eliminate log_2_ (*C*_•_*_i_*/*C*_•_*_s_*) from the statistical model, we use a normalizing covariate, *z_i_*. Many normalization methods can be implemented through an analysis of covariance (Parrish and Delongchamp, 2006). In the example, we normalize using housekeeping genes and the following details are for that case.

Define housekeeping genes, g ∈ ℋ, as a set of genes that satisfy *C_gi_* = *C_gj_* for all samples, i.e. genes which are not affected by the treatments. Let the normalizing covariate be the average of log-intensities for the housekeeping genes from a sample, i.e. $z_{i}=h^{-1}\sum_{g\in H}y_{gi}$. Define ${\text{log}}_{2}{\bar{C}}_{H}=h^{-1}\sum_{g\in H}{\text{log}}_{2}(C_{gi}/C_{gs})$ and note that this quantity is the same for all samples, *i* = 1,…,*n*, because of the *a priori* assumption that these are housekeeping genes. Equation (1.6) implies

(1.7) $$E(z_{i})=\text{E}\left[\bar{d}\right]+{\text{log}}_{2}{\bar{C}}_{H}-{\text{log}}_{2}(C_{•i}/C_{•s}).$$

By combining Equations (1.6) and (1.7), the expectation of an observed intensity is

(1.8) $$\text{E}(y_{gi})\approx X_{i}\beta_{g}+\text{E}\left[d-\bar{d}\right]-{\text{log}}_{2}{\bar{C}}_{H}+\text{E}(z_{i}).$$

This relationship is an analysis of covariance model,

(1.9) $$\text{E}\left[{\text{log}}_{2}I_{ga(i)}\right]=X_{i}\beta_{g}+\gamma_{g}z_{i},$$

where the location shift (bias) implied by E[*d*−*d̄*] − log_2_ *C̄*_ℋ_ is absorbed into the overall mean of the linear model, **X***_i_***β***_g_*, and the parameter, *γ*_g_, captures the attenuation associated with replacing the expectation, *E*(*z_i_*), with an estimate, *z_i_*.

This statistical model is an analysis of covariance model with two generic sources of variation, biological and technical. Biological variation should be estimated through replicate samples within treatments. Technical variation arises from sources connected with the sample-processing steps in the measurement of gene expression. These sources are associated with spots, dyes and batchs.

### Example

The example discusses the design of a toxicogenomics study to evaluate treatment effects on mRNA transcripts involved in mitochondrial function. It illustrates a situation where tissues are collected from a bioassay designed primarily for other purposes. In this case, a bioassay was conducted at NCTR for the National Toxicology Program, which is assessing the carcinogenic potential of AZT when it is administered to prevent mother to child transmission of AIDS. Muscle tissue was collected from 120 mice, 6 mice per treatment from 20 treatments: 2 sexes × 2 sacrifice times × 5 levels of AZT exposure. AZT is known to affect the mitochondria and the microarray assay is intended to complement an overall toxicity assessment by testing for treatment effects on transcript levels of 542 genes important to the structure and/or function of mitochondria (Desai et al. 2007).

The 20 ‘treatments’ in this bioassay are arranged in a factorial design of 5 AZT dosing regimes in both sexes with tissues collected at either a one or two hour interval after the final AZT exposure. This arrangement of treatments can be parameterized in several equivalent ways. Table 2 gives one parameterization, which serves as the basis of discussions to follow. Table 2 is a standard partitioning of the sums of squares into sources of variation, and it corresponds to a fixed effects linear model, Equation (1.1). The statistical significance of each source would be evaluated through an analysis of variance. In the context of measuring gene expression, this defines the statistical analysis of the data for an arbitrary gene if its mRNA concentrations in each mouse were measured with a simple additive error, i.e. Equation (1.1).

In the design we specifically want to account for biological variation associated with mice (*σ_m_*^2^) and technical variation associated with batches (σ*_b_*^2^), as well as spots and dyes. Effects of spots and dyes are eliminated by analyzing the difference between the log-intensities on the red and green channels. Variation (e.g. amount and uniformity of the spotting) associated with the probe is ‘removed’ by subtracting the CY5 log-intensity from the CY3 log-intensity. The sample is labelled with CY5 and a universal reference is labelled with CY3, which effectively removes dye effects from treatment contrasts (Dobbin et al. 2003a; Dobbin et al. 2003b).

In addition, each probe is spotted twice on the array providing a sub-sampling error. Conceptually, this source of variation is a lower bound on the technical variation which can be partitioned from the residual technical variation (*σ*^2^). This error can be quite useful for diagnostic purposes, and it is routinely evaluated at the analysis stage (Delong-champ et al. 2005). Because this level of detail does not alter the main design issues, this component was absorbed into the residual in the expected mean squares that are presented here. That is, the tabled expectations assume that the log-intensity differences for the two spots have been averaged.

The design has to accommodate two logistic constraints. First, the amount of mRNA that can be extracted reliably from each sample is anticipated to be near the minimum required for a microarray assay. We formally considered 2 options, 1) using 4 mice (assumes at least 4 among the 6 mice will have enough mRNA) or 2) pooling the mRNA from 2 mice so that the 6 mice form 3 mRNA samples. The second constraint is that the microarray laboratory can concurrently process 6 to 8 tissue samples on DNA arrays. Consequently, arrays for 20 treatments cannot be processed as a batch. Since the primary interest is in AZT effects, we chose to process 5 samples as a batch, one sample from each AZT treatment within a sacrifice time and sex.

Table 3 outlines the degrees of freedom and expected mean squares under the first option. Table 4 outlines the degrees of freedom and expected mean squares under the second option, where it is assumed that pooling 2 mice reduces the biological variance to σ*_m_*^2^/2. Note that the first option uses 80 arrays while the second option uses 60 arrays. Also the first option is more ‘iffy’ since one cannot guarantee that 4 of the samples will have enough mRNA for a microarray assay. Consequently, option 2 is preferred unless the statistical power under option 1 is substantially better.

It is not entirely clear how to evaluate the power of competing designs because of the large number of comparisons (Tsai et al. 2005). In this setting, the significance of a p-value is difficult to interpret. In our analyses, the genes are ordered by their p-value, and a gene with a smaller p-value is presumed to be more significant (Delongchamp et al. 2004). Significant genes are identified by a *post hoc* evaluation of the false discovery rate associated with any partition of the ordered genes into an ‘affected’ set and the remainder, presumed not to be affected. We implement this analysis strategy at the design stage by decreasing the significance level used in a conventional power computation. In the power calculation, we want to detect most of the genes that have sufficiently large treatment-induced changes, say greater than 1.5 fold. That is, if a gene has a 1.5-fold treatment-induced change, the statistical test should have a reasonably small p-value, say *p* ≈ 1/(# genes on the array), with a high probability (power). In this example where there are 542 mitochondrial genes on the array, we used *p* = 0.002 (1/500) for the power computations.

Obviously, power depends on the values of variance components in Tables 3 or 4, which are gene-specific and unknown (Tsai et al. 2005). Because so many important parameters are unknown, a comprehensive assessment of power is not attempted. For a ball park assessment of competing designs, the power of a two-sided t-test for an interesting contrast can be computed using the median values of variance components from a previous study. Typically, one must choose somewhat arbitrarily among available studies attempting to most closely match the array, tissue and mouse strain of the current study. In this case, data from an AZT study using the same array on liver samples from the same mouse strain were available (Desai et al. 2007). This study gave the following medians for variance components of the 542 mitochondrial genes on the array:

$$\begin{array}{l} \sigma^{2}+2\sigma_{m}^{2}+10\sigma_{b}^{2}=0.174,\\ \sigma^{2}+\sigma_{m}^{2}+10\sigma_{b}^{2}=0.15,\\ \sigma^{2}+2\sigma_{m}^{2}=0.073,\\ \sigma^{2}+\sigma_{m}^{2}=0.054. \end{array}$$

**Figure 1:**
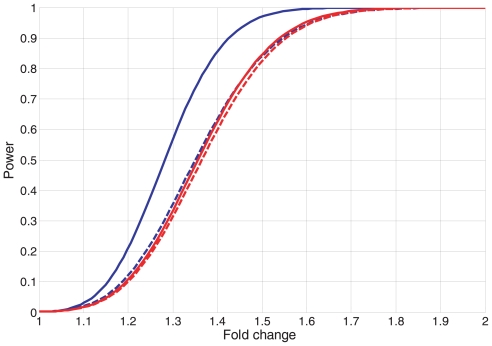
**Power versus fold change for two key contrasts.** The figure plots the power for a two-sided t-test at the 0.002 significance level as a function of the fold change. Blue lines give the power for a sex difference within a sacrifice time, and red lines give the power for a difference between two AZT levels within a sex and sacrifice time. Solid lines use the error for Option 1 (Table 3) and the dashed lines use the error for Option 2 (Table 4).

Under the factorial design (Tables 3 or 4), there are a number of hypotheses and sub-hypotheses that can be tested. We evaluate the overall performance of a design by specifying the power for a few contrasts, which we deem to be most important. Factorial designs have the most power to detect the main effects (in this case: ‘AZT’, ‘Sex’, and ‘Time’), but contrasts among levels of the main effects are not the most interesting when interactions are anticipated. In toxicological studies, the effect of a dosing regime is frequently believed to depend on the levels of other factors, which are included in the study to broaden the scope of the risk assessment. This is the case here where the mitochondrial toxicogenomics associated with AZT treatment may also depend on sex or sacrifice time (sacrifice times were based upon the pharmacokinetics of AZT metabolism in these mice). When interactions are significant, interpretations of the results depend on contrasts within levels of other factors. With this appraisal in mind, we evaluated the power for two ‘key’ contrasts, an AZT contrast within a sex and sacrifice time and a sex contrast within a sacrifice time. These contrasts use different error terms (Tables 3 or 4); the AZT contrast uses ‘Mice within Treatments’ and the sex contrast uses ‘Batch’. Figure 1 plots the power as a function of fold-change for these two contrasts under both design options. All four curves have adequate power to pick up 1.5 fold-changes at the 0.002 level of significance. Because both options have adequate power, option 2 was adopted as this design has better cost/logistic properties.

When substantial batch effects are present but ignored in the design and subsequent analyses, the computed F-ratios are unlikely to be valid tests of the nominal hypotheses. In general, the specific consequence of ignoring substantial batch effects would not be very tractable since they are unmanaged in the design. However for the design in Table 4, the batch effects are nested within sex and time effects and the result of disregarding batch effects can easily be evaluated. Under this design, proper analyses partition the variation as presented in Table 4. If an analyst were to disregard the ‘batches’ component such that this variation is simply added to the ‘mice within treatments’ component, then the pooled error term would have an expected mean squared error of approximately σ^2^ + σ_m_^2^ + 2σ_b_^2^, which would be estimated with 39 degrees of freedom. This ‘pooled’ estimate would misrepresent the appropriate error variances by over estimating the error variance of AZT treatments and under estimating the error variance of sex or time effects. The F-ratios would not be valid tests of the nominal hypotheses and would result in fewer AZT effects, more sex effects and more time effects than appropriate.

Several other designs are valid. They were not formally considered because they represent different priorities as to what effects are important. For example, an alternative is to process the arrays separately as ‘batches’ of one, in that sense ignoring batch. This design provides valid tests for the interesting factors by incorporating the variation associated with batches into the residual (Table 5). Compared to the design of Table 4, this design provides more precise estimates of the sex and time effects, but less precise estimates of AZT effects. Our preference for the design in Table 4 reflects our desire to estimate AZT effects, and an *a priori* belief that sex and time effects are less important. In addition, the design in Table 5 does not utilize the lab efficiently making this design more expensive. The lab is capable of processing six to eight arrays as a batch and this design processes one.

## Discussion

When DNA arrays are used to measure gene expression, several aspects of the technology should play a part in the experiment’s design. The observed intensities must be normalized for inferences to properly reflect treatment differences. Since normalization removes a major part of the variation, implementing the adjustment as a covariate gives a direct assessment of its impact. The assay of samples involves several steps and arrays processed concurrently produce measurements that are more homogeneous. Whenever samples are assayed in batches, the design should manage this effect. Likewise, when multiple (usually two) samples are hybridized to an array the measurements will be more homogeneous. Consequently, designs need to address variation from batches, dyes and arrays as well as the variation among samples (experimental units).

We view toxicogenomics studies as assays of mRNA in tissue samples nested within a bioassay of treatments. The purpose of the study is to estimate treatment differences in gene expression. Statistical tests of treatment effects require a valid assessment of within-treatment variation, which will generally be the variation among tissue samples from treated animals. As the example illustrates, tissue samples can be pooled but the design needs to estimate the variation among experimental units (pools in this example). The technical sources of variation associated with each mRNA assay (array) primarily need to be managed so that variance among experimental units can be estimated. We accomplish this by blocking on samples that are processed concurrently and samples measured on the same array.

As the example illustrates, samples often come from studies which were originally designed for reasons other than studying gene expression. In such cases, the design is constrained by the available samples. The use of standard samples adjusts simply for probe-specific effects and designs having valid tests, which adjust for batch, array, and dye, can be found for virtually any sample size.

Although background expression can be ignored at the design stage, some kind of strategy should address background at the analysis stage. A common strategy is to subtract an estimate of the background from the observed intensity. This strategy is not recommended for a couple of reasons. First, the usual estimates of background available to the analyst simply do not measure the appropriate background. Second, subtraction generates negative intensities which cannot be log-transformed and implemented in the planned analysis. In essence, our strategy is to only pursue treatment effects among those probes for which background expression is judged to be negligible (Delongchamp et al. 2005). Actually, all probes are analyzed but those with ‘background’ levels of expression for all samples are classified as ‘not expressed’. The results of analyses on these genes are separated from those for the ‘expressed’ genes. The ‘not expressed’ genes can and often do indicate problems with the statistical model (Delongchamp et al. 2004). Genes that are not expressed cannot be differentially expressed. So, the empirical distribution of p-values from tests of treatment effects should follow the null distribution. When this empirical distribution is not consistent with a uniform distribution, one of several potential problems is an inappropriate design.

Normalization is necessary and a difficult problem in practice. We have avoided the issues by assuming the existence of a normalizing covariate. This is a reasonable approach when developing an experiment’s design, but at the time of analysis one needs to produce a covariate. There are a number of methods which have been proposed for normalization and many can be implemented through an analysis of covariance (Parrish and Delongchamp, 2006). Normalization methods potentially mitigate effects more properly considered as batch effects or array effects in addition to addressing the adjustment discussed in this paper. We caution against relying on normalization adjustments in lieu of blocking because they can introduce large biases (Delongchamp and Kodell, 2004; Parrish and Delongchamp, 2006).

## Conclusions

Many toxicogenomics studies are bioassays which measure gene expression using DNA arrays. These studies can be designed and analyzed using standard methods with a few modifications to account for characteristics of array measurements, such as multiple endpoints and normalization. As much as possible, technical variation associated with probes, dyes, and batches are managed by blocking treatments within these sources of variation. An example shows how some practical constraints can be accommodated by this modelling and how it allows one to objectively compare competing designs.

## Figures and Tables

**Table 1:** **Dye flip between two samples.**Table defines a dye flip for two samples on two arrays. The log-intensities for dye *i* and array *j* are denoted *y_ij._*

	Array 1	Array 2
Dye: CY3	Sample *i: y*_11_	Sample j: *y*_12_
Dye: CY5	Sample *j: y*_21_	Sample *i: y*_22_

**Table 2:** **Expected mean squared errors for the bioassay.**Table shows the expected mean squared errors associated with a fixed-effects 2 × 2 × 5 factorial model for the factors: sex, time, AZT and their interactions. This layout corresponds to a specific parameterization satisfying Equation 1.1.

Source	DF	Expected mean square
Sex	1	*σ*^2^*_m_* + *Q*(Sex)
Time	1	*σ*^2^*_m_* + *Q*(Time)
Sex * Time	1	*σ*^2^*_m_* + *Q*(Sex * Time)
AZT	4	*σ*^2^*_m_* + *Q*(AZT)
AZT * Sex	4	*σ*^2^*_m_* + *Q*(AZT * Sex)
AZT * Time	4	*σ*^2^*_m_* + *Q*(AZT * Time)
AZT * Sex * Time	4	*σ*^2^*_m_* + *Q*(AZT * Sex * Time)
Mice within treatment	20 (*n* − 1)	*σ*^2^*_m_*

**Table 3:** **Expected mean squared errors for option 1 of the combined model.**Table shows the expected mean squared errors associated with a combined model. This layout corresponds to a 2 × 2 × 5 factorial arrangement of sex, time, AZT and their interactions with the 80 muscle samples processed in 16 batches (blocks); each batch has 5 samples, one sample for each AZT level within a sex and a sacrifice time.

Source	DF	Expected mean square (approx.)
Mean: Housekeeping genes	1	σ^2^ + 2σ^2^*_m_* + *Q*(Slope)
Sex	1	σ^2^ + 2σ^2^*_m_* + 10σ^2^*_b_* + *Q*(Sex)
Time	1	σ^2^ + 2σ^2^*_m_* + 10σ^2^*_b_* + *Q*(Time)
Sex * Time	1	σ^2^ + 2σ^2^*_m_* + 10σ^2^*_b_* + *Q*(Sex * Time)
Batch	12	σ^2^ + 2σ^2^*_m_* + 10σ^2^*_b_*
AZT	4	σ^2^ + 2σ^2^*_m_* + *Q*(AZT)
AZT * Sex	4	σ^2^ + 2σ^2^*_m_* + *Q*(AZT * Sex)
AZT * Time	4	σ^2^ + 2σ^2^*_m_* + *Q*(AZT * Time)
AZT * Sex * Time	4	σ^2^ + 2σ^2^*_m_* + *Q*(AZT * Sex * Time)
Mice within treatment	47	σ^2^ + 2σ^2^*_m_*

**Table 4:** **Expected mean squared errors for option 2 of the combined model.**Table shows the expected mean squared errors associated with a combined model. This layout corresponds to a 2 × 2 × 5 factorial arrangement of sex, time, AZT and their interactions with the 60 pooled muscle samples (2 samples per pool) processed in 12 batches (blocks); each batch has 5 pools, one pool for each AZT level within a sex and a sacrifice time.

Source	DF	Expected mean square (approx.)
Mean: Housekeeping genes	1	σ^2^ + σ^2^*_m_* + *Q*(Slope)
Sex	1	σ^2^ + σ^2^*_m_* + 10σ^2^*_b_* + *Q*(Sex)
Time	1	σ^2^ + σ^2^*_m_* + 10σ^2^*_b_* + *Q*(Time)
Sex * Time	1	σ^2^ + σ^2^*_m_* + 10σ^2^*_b_* + *Q*(Sex * Time)
Batch	8	σ^2^ + σ^2^*_m_* + 10σ^2^*_b_*
AZT	4	σ^2^ + σ^2^*_m_* + 10σ^2^*_b_* + *Q*(AZT)
AZT * Sex	4	σ^2^ + σ^2^*_m_* + 10σ^2^*_b_* + *Q*(AZT * Sex)
AZT * Time	4	σ^2^ + σ^2^*_m_* + 10σ^2^*_b_* + *Q*(AZT * Time)
AZT * Sex * Time Time)	4	σ^2^ + σ^2^*_m_* + 10σ^2^*_b_* + *Q*(AZT * Sex *
Mice within treatment	31	σ^2^ + σ^2^*_m_*

**Table 5:** **Expected mean squared errors for option 2 of the combined model with one array per batch.**Table shows the expected mean squared errors associated with a combined model. This layout corresponds to a 2 × 2 × 5 factorial arrangement of sex, time, AZT and their interactions with the 60 pooled muscle samples (2 samples per pool) processed in 60 batches (blocks); each batch has 1 pool.

Source	DF	Expected mean square (approx.)
Mean: Housekeeping genes	1	σ^2^ + σ^2^*_b_* + σ^2^*_m_* + *Q*(Slope)
Sex	1	σ^2^ + σ^2^*_b_* + σ^2^*_m_* + *Q*(Sex)
Time	1	σ^2^ + σ^2^*_b_* + σ^2^*_m_* + *Q*(Time)
Sex * Time	1	σ^2^ + σ^2^*_b_* + σ^2^*_m_* + *Q*(Sex * Time)
AZT	4	σ^2^ + σ^2^*_b_* + σ^2^*_m_* + *Q*(AZT)
AZT * Sex	4	σ^2^ + σ^2^*_b_* + σ^2^*_m_* + *Q*(AZT * Sex)
AZT * Time	4	σ^2^ + σ^2^*_b_* + σ^2^*_m_* + *Q*(AZT * Time)
AZT * Sex * Time	4	σ^2^ + σ^2^*_b_* + σ^2^*_m_* + *Q*(AZT * Sex * Time)
Mice within treatment	39	σ^2^ + σ^2^*_b_* + σ^2^*_m_*
